# Downregulation of long non‐coding RNA LOC101928477 correlates with tumor progression by regulating the epithelial‐mesenchymal transition in esophageal squamous cell carcinoma

**DOI:** 10.1111/1759-7714.13858

**Published:** 2021-03-13

**Authors:** Demiao Kong, Dali Long, Bo Liu, Dengke Pei, Na Cao, Guihua Zhang, Zhenkun Xia, Meng Luo

**Affiliations:** ^1^ Department of Thoracic Surgery Guizhou Provincial People's Hospital Guiyang China; ^2^ Department of Intensive Care Unit Guizhou Provincial People's Hospital Guiyang China; ^3^ Department of Logistics Guizhou Provincial People's Hospital, Guizhou Guiyang China

**Keywords:** epithelial‐mesenchymal transition, esophageal squamous cell carcinoma, LOC101928477

## Abstract

**Background:**

Esophageal squamous cell carcinoma (ESCC) is one of the deadliest malignancies. There is a growing body of evidence showing that long non‐coding RNAs (lncRNAs) play critical roles in ESCC oncogenesis. The present study aimed to explore the role of LOC101928477, a newly discovered lncRNA, in the development and metastasis of ESCC.

**Methods:**

In this study, real‐time PCR, western blotting, cell counting kit‐8 (CCK‐8), flow cytometry, colony formation, wound healing, transwell migration/invasion assay, immunofluorescence, and immunohistochemistry were used. We also applied an in situ xenograft mouse model and a lung metastasis mouse model to verify our findings.

**Results:**

We determined that LOC101928477 expression was inhibited in ESCC tissue and ESCC cell lines when compared with controls. Moreover, forced expression of LOC101928477 effectively inhibited ESCC cell proliferation, colony formation, migration, and invasion via suppression of epithelial‐mesenchymal transition (EMT). Furthermore, LOC101928477 overexpression inhibited in situ tumor growth and lung metastasis in a mouse model.

**Conclusions:**

Together, our results suggested that LOC101928477 could be a novel suppressor gene involved in ESCC progression.

## Graphical abstract

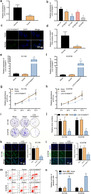

## INTRODUCTION

Esophageal squamous cell carcinoma (ESCC) is considered to be one of the deadliest malignancies worldwide.^1^, ^2^, ^3^, ^4^ It has previously been reported that the lifetime risk of ESCC is 0.75 and 0.26% in males and females, respectively.^5^ In addition, the incidence of ESCC continues to rise rapidly each year.^5^ So far, the prognosis of ESCC patients is still extremely poor^6^ and the underlying molecular features of ESCC invasion and metastasis remains to be elucidated.^7^, ^8^

Long non‐coding RNA (lncRNA) is commonly an RNA molecule >200 nucleotides in length, which is not translated into a protein.^9^, ^10^, ^11^ Recent evidence shows that lncRNAs are involved in the regulation of gene expressions in both antitumor and oncogenic pathways in a variety of human cancers such as ESCC.^12^, ^13^, ^14^, ^15^ lncRNAs can effectively influence ESCC cell growth, survival, migration/invasion, and metastasis.^11^, ^16^, ^17^ For example, lncRNA ATB has been reported to promote malignancy of ESCC by regulating the miR‐200b/Kindlin‐2 axis.^18^ Upregulation of lncRNA CASC9 has been shown to promote ESCC growth by negatively regulating PDCD4 expression through EZH2.^15^ Also, lncRNA SPRY4‐IT1 has been reported to promote the metastasis of ESCC via induction of the EMT, again indicating that lncRNAs may play critical roles in ESCC oncogenesis.^19^

LOC101928477, a newly discovered lncRNA, is located on chromosome 11, next to the MMP10 gene. By conducting bioinformatic analyses and RNA‐sequencing, LOC101928477 has been found to be significantly downregulated in ESCC tissue, indicating a possible role of LOC101928477 in regulating the development of ESCC.^20^ However, the biological functions of LOC101928477 are currently mostly unknown.

The present study aimed to examine the clinical significance of LOC101928477 in ESCC and to identify its biological functions on proliferation, migration, and the EMT of ESCC cells. We hypothesized that LOC101928477 could be a novel suppressor gene involved in ESCC progression.

## METHODS

### Clinical sample collection

This study was approved by the Medical Ethics Committee of Guizhou Provincial People's Hospital (No. 201710323), as formulated based on the Declaration of Helsinki. Informed consent was given by all patients. Clinical ESCC samples (*n* = 30) and matching noncancerous esophageal tissue (*n* = 30) were collected during esophageal cancer resection surgery and frozen in liquid nitrogen. None of the patients received chemo‐ or radiotherapy prior to surgery. The baseline characteristics of the patients included in the study are presented in Table 1.

**TABLE 1:** Clinicopathological patient characteristics

Clinical parameter	No. of patients (*n* = 30)
Gender	
Female	14
Male	16
Age	
Median	55.43
Range	39–77
Tumor size	
<4 cm	19
≥4 cm	11
Distant metastasis	
Yes	0
No	30

### Cell culture and transfection

Human ESCC cell lines (EC109, EC9706, KYSE30, KYSE450) and normal human esophageal epithelial cell line Het‐1A were obtained from the Cell Bank of the Chinese Academy of Sciences. All cells were cultured in RPMI‐1640 medium (Hyclone) supplemented with 10% fetal bovine serum (FBS; HyClone) in a humidified atmosphere at 37°C with 5% CO_2_. To overexpress LOC101928477, cells (EC109 and EC9706) were seeded on six‐well plates at a density of 3 × 10^5^ and full‐length coding sequence of LOC101928477 was amplified and subcloned into the pcDNA^3.1(+)^ vector (Invitrogen) according to the manufacturer's instructions.^21^, ^22^, ^23^ Cells were untransfected (mock group) or transfected with a negative control vector (vector group) or the LOC101928477‐overexpressing plasmid (LOC101928477 group) following the standard protocol. siRNA targeting MMP10 were prepared by GenePharma.

### Real‐time PCR

Total RNA was isolated from tissue or cells using TRIzol reagent (Invitrogen) and was then reverse‐transcribed into cDNA using PrimeScript real‐time PCR kit (TaKaRa Biotechnology). The real‐time PCR amplification was performed using a SYBR Green PCR Master Mix (TaKaRa) on the ABI 7900 Prism HT (Applied Biosystems). GAPDH was used to internally normalize the expression level of each candidate gene. The relative quantitative comparison between groups was evaluated using the 2^−ΔΔCt^ method.^15^, ^24^, ^25^, ^26^ Primers for real‐time PCR were as follows: LOC101928477: forward 5′‐CTGCAAGTTTGGGGGTGAAC‐3′, reverse 5′‐ACTGTGGCGAGGTGATATGG‐3′, GAPDH forward 5′‐GGAGCGAGATCCCTCCAAAAT‐3′, reverse 5′‐GGCTGTTGTCATACTTCTCATGG‐3′.

### Cell viability assay

Cell proliferation was measured with a cell counting kit‐8 (CCK‐8; Dojindo). Briefly, the cells were seeded in 96‐well culture plates at a density of 5 × 10^3^/well. Cell viability was then assessed using the CCK8 at 24, 48, and 72 h after transfection by measuring the absorbance at 570 nm^3^.

**Flow cytometry**

EC109 and EC9706 cells were seeded at a density of 3 × 10^5^ cells/well in six‐well plates. Then, 48 h after transfection, EC109 and EC9706 cells were harvested by trypsinization. Apoptotic assay (BD Pharminge FITC‐Annexin V Apoptosis Detection Kit [BD Biosciences]) was used to perform flow cytometry. Apoptotic rates of each sample were confirmed with a flow cytometer (ACSCalibur, Becton Dickinson).^3^, ^26^, ^27^, ^28^

### Fluorescence in situ hybridization analysis (FISH)

A pool of RNA FISH probes against LOC101928477 was designed and synthesized using Stellaris probe designer program according to the manufacturer's instructions.^15^, ^29^ The air‐dried cells were incubated overnight at 37°C in 2 × SSC, 10% formamide and 10% dextran to carry out the hybridization. After being washed and dehydrated, the slides were mounted with Prolong Gold Antifade Reagent with DAPI for detection of nucleic acids. Cells on the slides were visualized for FISH using a fluorescence microscope (DMI4000B, Leica).^29^

### Immunofluorescence (IF)

EC109 and EC9706 cells were seeded onto sterile coverslips (WHB‐24‐CS, diameter: 14 mm) at a density of 5 × 10^5^ cells/coverslip and incubated for 4 h. Cells were then fixed with 4% formaldehyde and coverslips with fixed cells incubated overnight at 4°C with antibodies specific to E‐cadherin (1:100; Abcam), N‐cadherin (1:100; Abcam), Ki‐67 (1:100; Abcam) and MMP10 (1:100; Abcam). Fluorescence‐conjugated secondary antibodies (1:100; Beyotime) were used for 2 h at room temperature. DAPI (Beyotime, China) was used to stain the nuclei.^3^, ^29^

### Immunohistochemistry

Tumors from mice were stained with IHC for Ki‐67 and MMP10. Briefly, specimens were incubated overnight at 4°C with antibodies specific to Ki‐67 (1:100; Abcam) and MMP10 (1:100; Abcam) in an avidin‐biotin complex method. The signal was later amplified and visualized with a secondary antibody (Beyotime), followed by 3′‐diaminobenzidine chromogen.^7^, ^30^, ^31^

### Colony formation

EC109 and EC9706 cells were seeded into six‐well plates at a density of 1 × 10^3^ cells/well and incubated for two weeks. The cells were then washed twice with PBS, fixed with 4% methanol, and stained with crystal violet. The number of colonies containing ≥50 cells were counted under a microscope (DM IL, Leica Microsystems).^16^, ^23^, ^32^

### Wound healing

EC109 and EC9706 cells were seeded into six‐well plates and scratched using a 1 ml tip after cells formed a confluent monolayer.^33^ The closure of the wound was analyzed under a microscope (DM IL, Leica Microsystems), and images were collected at 0 and 24 h. Image J software was utilized to quantify and compare the relative migration rate between groups.

## MIGRATION AND INVASION

For transwell analysis, EC109 and EC9706 cells (1.0 × 10^5^) in 100 μl of serum‐free medium were incubated for the (non‐) Matrigel‐coated upper chamber (8 μm pore size, BD Falcon cell culture inserts, BD Biosciences). Then, 500 μl of 10% FBS containing medium was added to the lower chamber. After 24 h incubation, nonmigrated/invaded cells on the upper surface were removed with a cotton swab and cells migrated to the lower chamber were fixed with 4% methanol and stained with crystal violet. The number of migrated cells was recorded under a microscope (DM IL, Leica Microsystems) in five random fields and calculated as the average per field.^2^, ^7^, ^31^

### Western blotting

Cells were lysed using RIPA lysis buffer (Beyotime) containing a protease inhibitor cocktail (Beyotime). After measuring the protein concentration (BCA assay), protein extracts were separated by 10% SDS‐PAGE and transferred onto PVDF (Pierce, Rockford). Following blocking with 5% nonfat milk, the membranes were incubated with antibodies specific to E‐cadherin (1:100; Abcam), N‐cadherin (1:100; Abcam), and MMP10 (1:100; Abcam) overnight at 4°C. Then, HRP‐conjugated secondary antibodies (1:5000, Boster) were applied for 2 h at room temperature. GAPDH (1:5000, Proteintech) was used as control. The results were quantified by Quantity One software (Bio‐Rad).^11^, ^26^, ^29^, ^34^

### Xenograft tumor growth model

All animal experiments were approved following the Guide for the Laboratory Animal Care and Use Committee of Guizhou Provincial People's Hospital (No. 201710324). BALB/c nude mice (4–5‐week‐old females) were recruited from Shanghai Experimental Animal Center. EC109 cells transfected with LOC101928477‐overexpressing plasmid or negative control were harvested and injected into the right side of the posterior flank of the BALB/c nude mice at a density of 5 × 10^5^/mouse (*n* = 6). Tumor volumes were calculated every four days using the equation: Volume = (length × width^2^)/2. At the end of the experiment (24 days post injection), the mice were sacrificed, and tumors were collected.^23^, ^35^, ^36^

#### Metastasis assays

Briefly, EC109 cells (1 × 10^6^ cells in PBS) transfected with LOC101928477‐overexpressing plasmid or negative control were injected through the lateral tail vein of nude mice. Thirty‐five days later, the lungs were collected and kept in liquid nitrogen or fixed in formalin for further analysis. The metastatic lung nodules were examined and counted.^9^, ^36^, ^37^

### Statistical analysis

All experiments were conducted at least three times in this study. SPSS 20.0 software (SPSS, Inc.) was used. Significant differences were calculated using Student's two‐tailed *t*‐test or one‐way ANOVA with Dunnett's test/Newman Keuls test. A *p*‐value < 0.05 was considered statistically significant.

## RESULTS

### LOC101928477 expression is inhibited in ESCC tissue and ESCC cell lines

**FIGURE 1:**
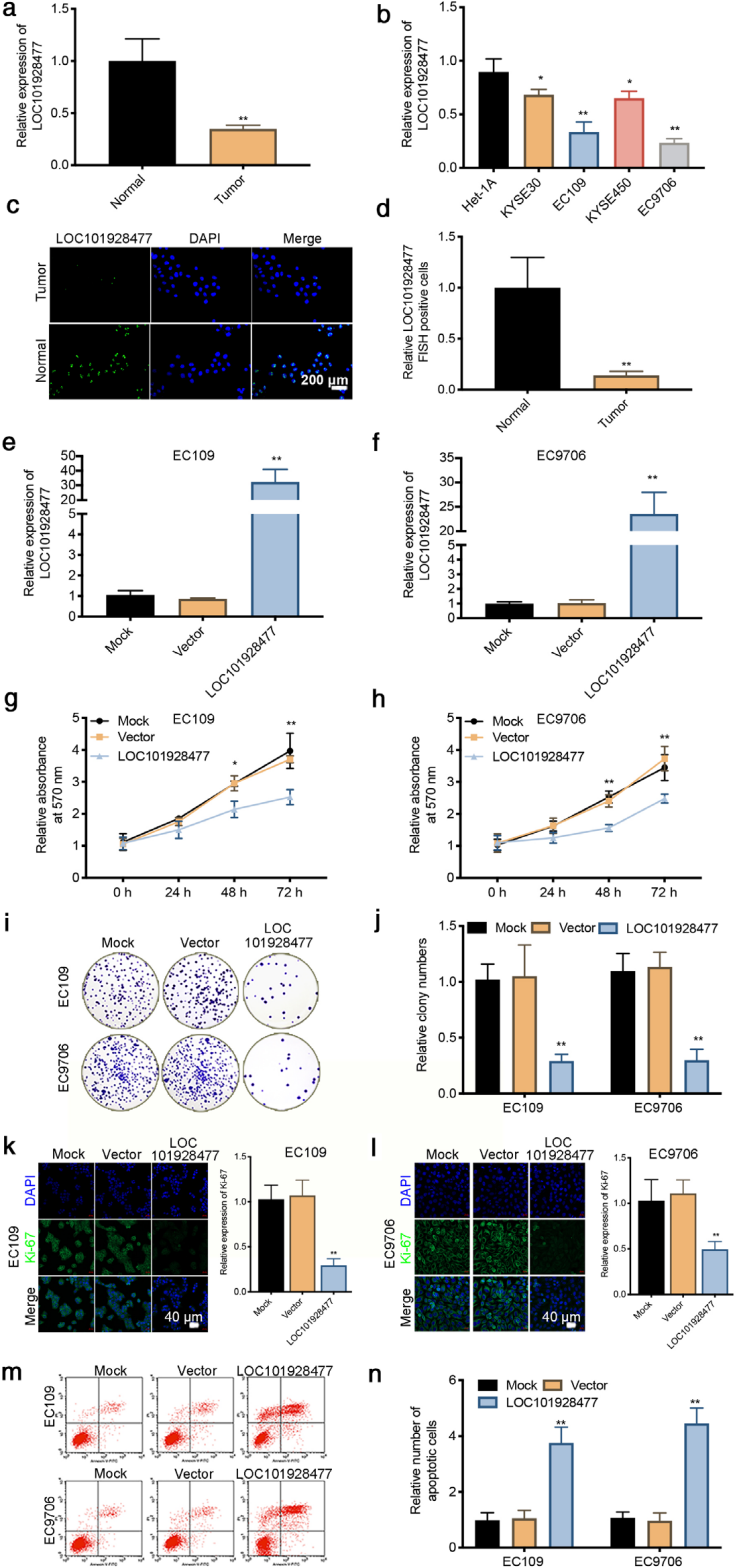
LOC101928477 inhibits esophageal squamous cell carcinoma (ESCC) cell proliferation. (a) ESCC tissue showed lower LOC101928477 mRNA expression when compared with paired normal tissue. (b) The mRNA expression level of LOC101928477 in ESCC cells (especially EC109 and EC9706) were significantly lower than that of the normal human esophageal epithelial cell line. (c, d) The results of FISH confirmed that LOC101928477 was expressed in the nuclei and was consistently less enriched in the nuclei of cells from ESCC tissue compared to cells from adjacent nontumor tissue. Scale bar: 200 μm.(e, f) Successful overexpression of LOC101928477 in both EC109 and EC9706 cells. (g, h) CCK‐8 assay showed that LOC101928477 inhibited ESCC cell proliferation. (i, j) Plate colony formation assay showed fewer colonies in LOC101928477‐ overexpressed group. (k–l) LOC101928477 inhibited the expression of Ki‐67. Scale bar: 40 μm. (m–n) LOC101928477 increased the apoptosis of EC109 and EC9706 cells. *p < 0.05, **p < 0.01 compared with the results of the control groups

We found that ESCC tissue had a significantly decreased LOC101928477 mRNA expression (Figure 1a) and the mRNA expression level of LOC101928477 in ESCC cells was also lower than that of the normal human esophageal epithelial cells (Figure 1b). The results of FISH confirmed that LOC101928477 was expressed in the nuclei and was consistently less enriched in the nuclei of cells from ESCC tissue, compared

with cells from adjacent nontumor tissue (Figure 1c,d). In addition, ESCC tissue showed higher MMP‐10 and Ki‐67 expression when compared with paired normal tissue. Western blot analyses showed that the protein expression level of MMP‐10 in ESCC cells (especially EC109 and EC9706) were significantly higher than that of the normal human esophageal epithelial cell line (Figure S1a,b).

### LOC101928477 inhibits ESCC cell proliferation

Based on the LOC101928477 mRNA expression level mentioned above, EC109 and EC9706 cells were chosen to investigate the effects of LOC101928477 on ESCC. We first confirmed the successful overexpression of LOC101928477 (Figure 1e, f). The CCK‐8 assay showed that LOC101928477 inhibited the proliferation of EC109 and EC9706 cells (Figure 1g,h). Similarly, plate colony formation assay showed fewer colonies in LOC101928477 overexpressed cells (Figure 1i, j). Furthermore, Figure 1k,l showed that LOC101928477 inhibited the expression of Ki‐67, a prognostic parameter of most cancers, and increased ESCC cell apoptosis (Figure 1m, n).

### LOC101928477 inhibits ESCC cell migration and invasion

**FIGURE 2:**
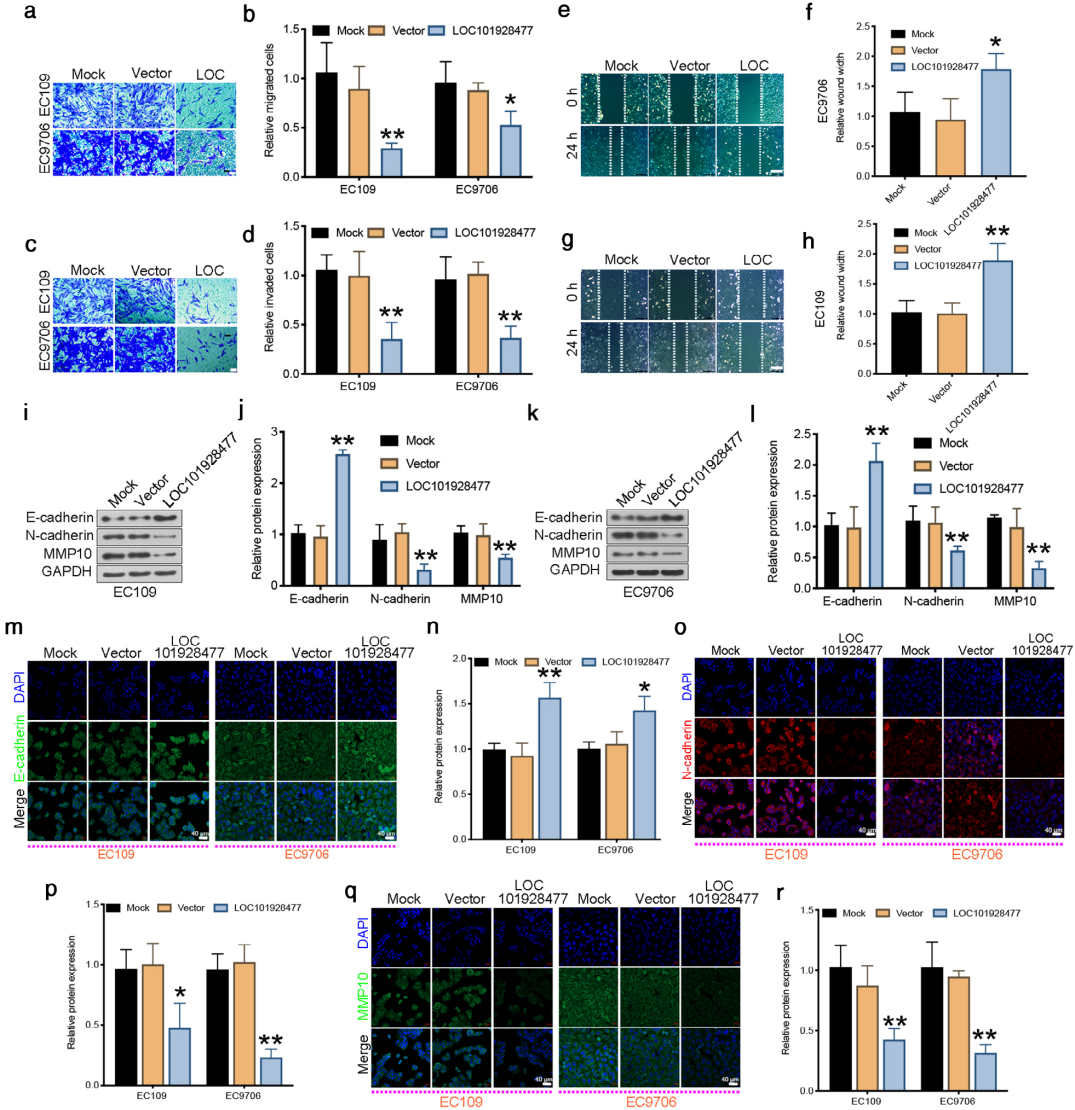
LOC101928477 inhibits the epithelial‐mesenchymal transition (EMT) in esophageal squamous cell carcinoma (ESCC) cells. (a, b) The results of the transwell migration assay. (c, d) Results of the invasion assay. (e–h) The wound healing assay. Scale bar (a,c): 50 μm; scale bar (e, g): 200 μm. (i–l) Western blot analysis of EC109 and EC9706 cells. (m–n) Immunofluorescence (IF) analysis showed that LOC101928477 increased the expression of E‐cadherin. (o, p) LOC101928477 inhibited the expression of N‐cadherin. (q, r) LOC101928477 in EC109 and EC9706 cells inhibited the expression of MMP10. Scale bar (m,o,q): 40 μm. **p < 0.01 compared with the results of the mock groups

The results of the migration (Figure 2a, b) and invasion assays (Figure 2c, d) showed that LOC101928477 decreased the migration and invasion ability of ESCC cells, which was consistent with the results of the wound healing assay (Figure 2e–h). In addition, plate colony formation assay showed more colonies in the LOC101928477‐knockdown group. Moreover, LOC101928477‐knockdown increased the wound‐healing capacity of ESCC cells (Figure S1c–e).

### LOC101928477 inhibits EMT

**FIGURE 3:**
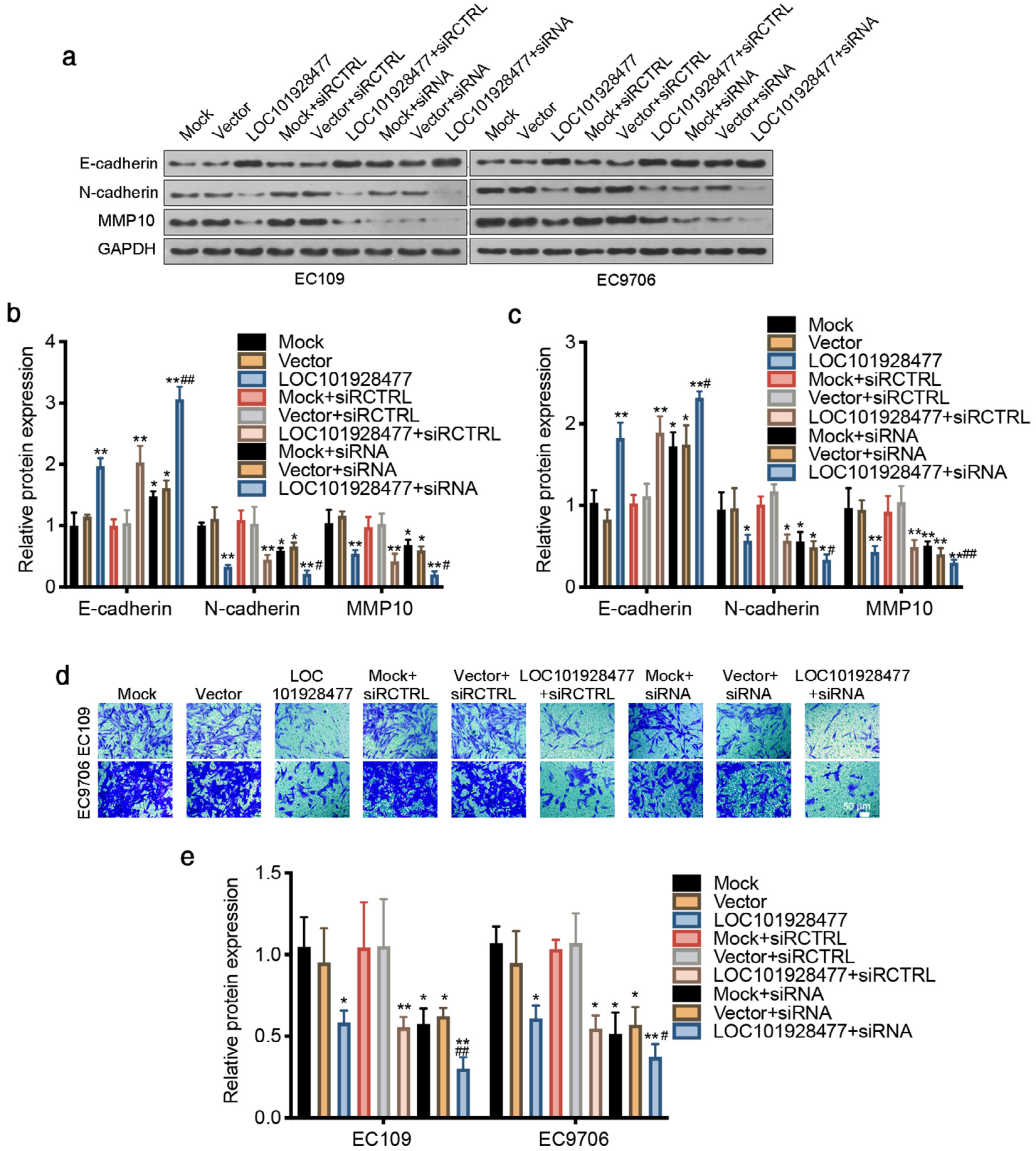
LOC101928477 suppresses epithelial‐mesenchymal transition (EMT) by targeting MMP‐10 (a–c) Interfering MMP10 enhanced the enrichment of E‐cadherin and the decrease of N‐cadherin and MMP10. (d, e) Interfering MMP10 enhanced the promoter effect of LOC101928477 on the transwell migration ability of EC109 and EC9706 cells. *p < 0.05, **p < 0.01 compared with the results of the Mock groups. ^**#**^ *p* < 0.05, ^**##**^ *p* < 0.01 compared with the results of the LOC101928477 groups. Scale bar: 50 μm

LOC101928477 decreased the expression of N‐cadherin and MMP10 *(*Figure 2i–l) and increased the expression of E‐cadherin in comparison with the control groups, which was confirmed by IF experiments (Figure 2m–r). siRNA interference of MMP10 was shown to enhance the enrichment of E‐cadherin and the decrease of N‐cadherin and MMP10 (Figure 3a–c). Moreover, MMP10 interference also enhanced the suppression of LOC101928477 on ESCC cell migration (Figure 3d,e). Together, these results indicated that overexpression of LOC101928477 partly blocked the EMT.

### LOC101928477 inhibits ESCC growth and metastasis

**FIGURE 4:**
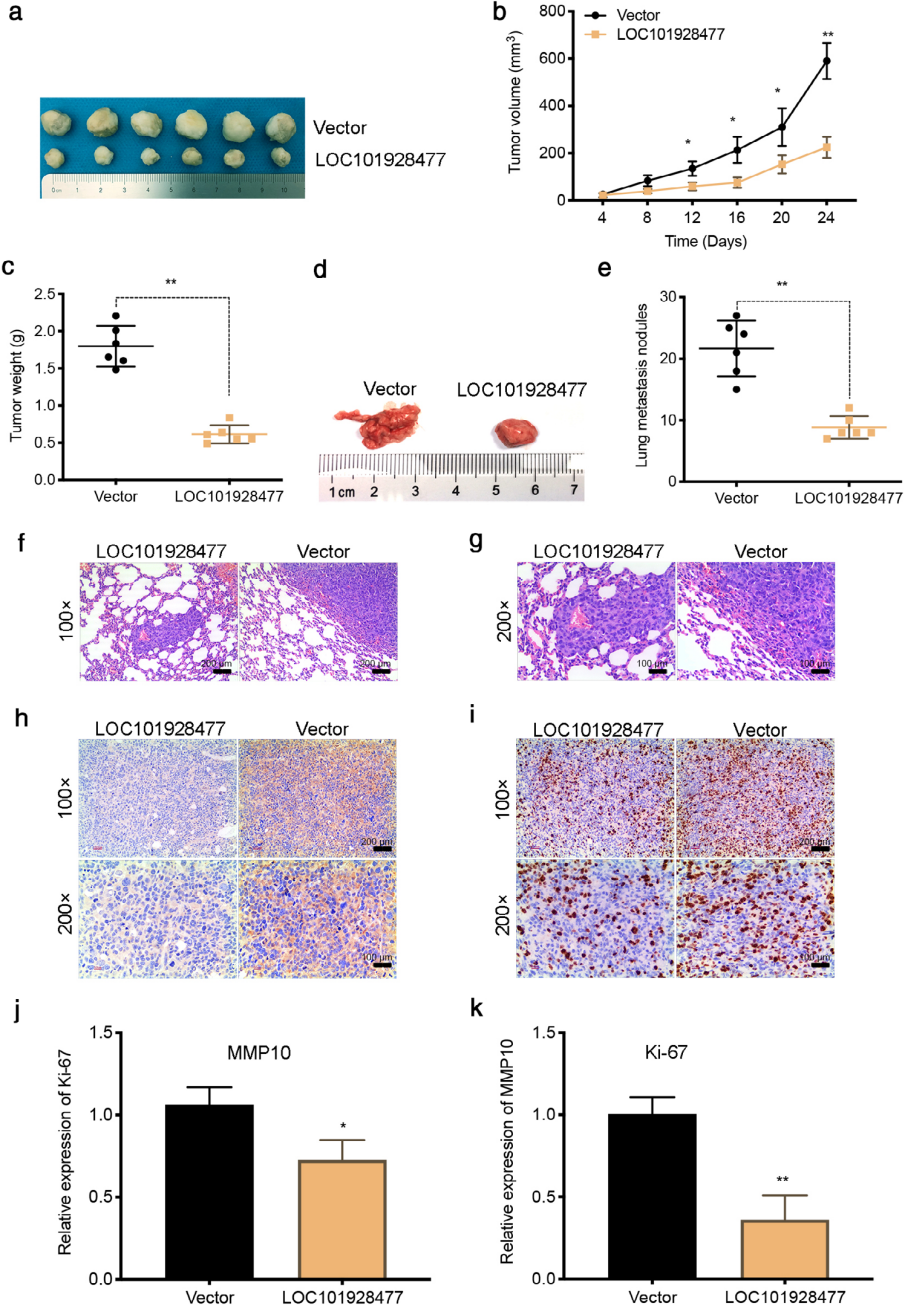
LOC101928477 inhibits esophageal squamous cell carcinoma (ESCC) cell growth and metastasis. (a–c) LOC101928477 reduced the volume and weight of tumors compared with the mice in the control group. (d, e) The tumor metastasis assay showed that fewer metastatic nodules in the lungs were observed by the LOC101928477‐overexpressed group when compared with those in the control group. (f, g) Hematoxylin & eosin (H&E) staining showed more tumor tissue in the control group than the LOC101928477 overexpression group. (h, i) Immunohistochemical (IHC) staining showed that LOC101928477 overexpression reduced the expression of MMP10 and Ki‐67 in tumor samples. *p < 0.05, **p < 0.01 compared with the results of the vector groups

LOC101928477‐overexpressed EC109 cells and the negative control (vector group) EC109 cells were injected into the right flank or the tail vein of nude mice. Figures 4a–c showed that LOC101928477 reduced the volume and weight of tumors. Furthermore, metastasis assay showed that fewer metastatic lung nodules were observed in the LOC101928477‐overexpressed group (Figure 4d,e). The result of H&E staining showed more tumor tissue in the control group (Figure 4f,g). LOC101928477 was also found to reduce the expression of Ki‐67 (Figure 4h,j) and MMP10 (Figure 4i,k).

## DISCUSSION

The focus of this study was to examine the clinical significance of LOC101928477 in ESCC and to identify its biological functions on proliferation, migration, and the EMT of ESCC cells. In this study, the expression of LOC101928477,^20^ was found to be downregulated in ESCC tissue and ESCC cell lines. Overexpression of LOC101928477 effectively inhibited ESCC cell proliferation, colony formation, migration, and invasion via suppression of the EMT. Moreover, LOC101928477 also inhibited ESCC tumor growth and lung metastasis in a xenograft model.

This study emphasized the role of LOC101928477 in EMT and ESCC metastasis. EMT has been widely accepted as a critical factor in tumor initiation and metastasis.^9^, ^26^, ^35^, ^37^ Its key regulatory role in invasion and metastasis has been reported in many human cancers including ESCC.^25^, ^28^, ^34^, ^38^, ^39^ EMT has also been connected to the induction of immunosuppression and drug resistance.^27^, ^40^ Furthermore, numerous clinical studies have also reported frequent downregulations of E‐cadherin and upregulations of N‐cadherin in ESCC patients.^27^ Studies have also suggested that therapeutics that target EMT inhibition may inhibit metastasis.^11^, ^30^, ^33^ In this study, LOC101928477 was proved to be capable of inhibiting EMT. Based on the in vitro results, LOC101928477 also markedly inhibited ESCC tumor growth and lung metastasis in vivo. The effectiveness of LOC101928477 found in regulating ESCC may be on the basis of the EMT activity.

The functions of lncRNAs are determined by its subcellular localizations. Nuclear lncRNAs have been reported to play roles in gene transcription. In the present study, LOC101928477 is a nuclear lncRNA located on chromosome 11, next to the MMP10 gene.^20^ As an important member of the matrix metalloproteinase (MMP) superfamily, MMP10 has been widely considered as a prognostic marker in various malignancies including ESCC. Activation of MMP10 has been previously reported to stimulate cancer cell proliferation, migration and metastasis.^31^, ^41^ In the present study, we found that the expression MMP10 was remarkably inhibited by LOC101928477, which was consistent with reduced tumor growth and metastasis. Thus, we believe that the effect of LOC101928477 on EMT and ESCC metastasis may occur, at least partially, through the inhibition of MMP10.

Due to the high propensity of metastasis, the prognosis of ESCC remains poor.^2^, ^8^, ^42^ The discovery of lncRNAs has recently emerged as a new and promising regulator of oncogenesis in many human cancers including ESCC.^24^, ^32^, ^36^, ^43^ We believe that this is the first study to characterize the biological function of LOC101928477, which may prove to be a novel therapeutic target in the future. However, we also acknowledge that the expression of LOC101928477 in ESCC tissue was first reported by Dai et al.,^20^ which may decrease the innovation of our study. Also, although we found that MMP10 expression was inhibited following LOC101928477 overexpression, the direct interactions between them were not studied. Thus, future studies are needed.

In summary, the results of the present study demonstrate that LOC101928477 inhibits tumor progression by regulating the epithelial‐mesenchymal transition in ESCC. Our results suggested that LOC101928477 could be a novel suppressor involved in ESCC progression.

## CONFLICT OF INTEREST

This research was supported by the National Natural Science Foundation of China (NSFC, No. 81301810) and Guizhou High‐level Talents Training Program (GZSYQCC[2014]003) . The authors declare no conflicts of interest.

## Supporting information


**Figure S1** Additional experiments using ESCC specimens and ESCC cells. (A) ESCC tissue showed higher MMP‐10 and Ki‐67 expression when compared with paired normal tissue. (B) Western blot analyses showed that the protein expression level of MMP‐10 in ESCC cells (especially EC109 and EC9706) was significantly higher than that of the normal human esophageal epithelial cell line. (C) Plate colony formation assay showed more colonies in LOC101928477‐knockdown group. (D‐E) LOC101928477‐knockdown increased the wound‐healing capacity of ESCC cells. Magnification: 100x.

## References

[1] Wen SW , Zhang YF , Li Y , Xu YZ , Li ZH , Lü H , et al. Isoalantolactone inhibits esophageal squamous cell carcinoma growth through downregulation of microRNA‐21 and derepression of PDCD4. Dig Dis Sci. 2018;63:2285–93. 29781054 10.1007/s10620-018-5119-z

[2] Liu RM , Sun DN , Jiao YL , Wang P , Zhang J , Wang M , et al. Macrophage migration inhibitory factor promotes tumor aggressiveness of esophageal squamous cell carcinoma via activation of Akt and inactivation of GSK3beta. Cancer Lett. 2018;412:289–96. 29079416 10.1016/j.canlet.2017.10.018

[3] Zhu H , Song H , Chen G , Yang X , Liu J , Ge Y , et al. eEF2K promotes progression and radioresistance of esophageal squamous cell carcinoma. Radiother Oncol. 2017;124(3):439–47. 28431753 10.1016/j.radonc.2017.04.001

[4] Yu X , Liang Q , Liu W , Zhou L , Li W , Liu H . Deguelin, an Aurora B kinase inhibitor, exhibits potent anti‐tumor effect in human esophageal squamous cell carcinoma. EBioMedicine. 2017;26:100–11. 29129699 10.1016/j.ebiom.2017.10.030 PMC5832566

[5] Demeester SR . Epidemiology and biology of esophageal cancer. Gastrointest Cancer Res. 2009;3(Suppl 2):S2–5. PMC2684731 19461918

[6] Guohong Z , Min S , Duenmei W , Songnian H , Min L , Jinsong L , et al. Genetic heterogeneity of oesophageal cancer in high‐incidence areas of southern and northern China. PLoS One. 2010;5:e9668. 20300624 10.1371/journal.pone.0009668 PMC2837742

[7] Wang L , Hou Z , Hasim A , Abuduerheman A , Zhang H , Niyaz M , et al. RNF113A promotes the proliferation, migration and invasion, and is associated with a poor prognosis of esophageal squamous cell carcinoma. Int J Oncol. 2018;52:861–71. 29393393 10.3892/ijo.2018.4253

[8] Tamaoki M , Komatsuzaki R , Komatsu M , Minashi K , Aoyagi K , et al. Multiple roles of single‐minded 2 in esophageal squamous cell carcinoma and its clinical implications. Cancer Sci. 2018;109(4):1121–34. 29427302 10.1111/cas.13531 PMC5891185

[9] Zheng X , Zhang Y , Liu Y , Fang L , Li L , Sun J , et al. HIF‐2alpha activated lncRNA NEAT1 promotes hepatocellular carcinoma cell invasion and metastasis by affecting the epithelial‐mesenchymal transition. J Cell Biochem. 2018;119(4):3247–56. 29091312 10.1002/jcb.26481

[10] Kong YG , Cui M , Chen SM , Xu Y , Xu Y , Tao ZZ . LncRNA‐LINC00460 facilitates nasopharyngeal carcinoma tumorigenesis through sponging miR‐149‐5p to up‐regulate IL6. Gene. 2018;639:77–84. 28987345 10.1016/j.gene.2017.10.006

[11] Zhou M , Zhang XY , Yu X . Overexpression of the long non‐coding RNA SPRY4‐IT1 promotes tumor cell proliferation and invasion by activating EZH2 in hepatocellular carcinoma. Biomed Pharmacother. 2017;85:348–54. 27899259 10.1016/j.biopha.2016.11.035

[12] Liang Y , Chen X , Wu Y , Li J , Zhang S , Wang K , et al. LncRNA CASC9 promotes esophageal squamous cell carcinoma metastasis through upregulating LAMC2 expression by interacting with the CREB‐binding protein. Cell Death Differ. 2018;25(11):1980–95. 29511340 10.1038/s41418-018-0084-9 PMC6219493

[13] Cui Y , Wu W , Lv P , Zhang J , Bai B , Cao W . Down‐regulation of long non‐coding RNA ESCCAL_1 inhibits tumor growth of esophageal squamous cell carcinoma in a xenograft mouse model. Oncotarget. 2018;9(1):783–90. 29416654 10.18632/oncotarget.23153 PMC5787510

[14] Chen Z , Lin J , Wu S , Xu C , Chen F , Huang Z . Up‐regulated miR‐548k promotes esophageal squamous cell carcinoma progression via targeting long noncoding RNA‐LET. Exp Cell Res. 2018;362(1):90–101. 29126868 10.1016/j.yexcr.2017.11.006

[15] Wu Y , Hu L , Liang Y , Li J , Wang K , Chen X , et al. Up‐regulation of lncRNA CASC9 promotes esophageal squamous cell carcinoma growth by negatively regulating PDCD4 expression through EZH2. Mol Cancer. 2017;16(1):150. 28854977 10.1186/s12943-017-0715-7 PMC5577767

[16] Zhu W , Zhuang P , Song W , Duan S , Xu Q , Peng M , et al. Knockdown of lncRNA HNF1A‐AS1 inhibits oncogenic phenotypes in colorectal carcinoma. Mol Med Rep. 2017;16(4):4694–700. 28791380 10.3892/mmr.2017.7175 PMC5647038

[17] Zhu G , Wang S , Chen J , Wang Z , Liang X , Wang X , et al. Long noncoding RNA HAS2‐AS1 mediates hypoxia‐induced invasiveness of oral squamous cell carcinoma. Mol Carcinog. 2017;56(10):2210–22. 28485478 10.1002/mc.22674

[18] Li Z , Wu X , Gu L , Shen Q , Luo W , Deng C , et al. Long non‐coding RNA ATB promotes malignancy of esophageal squamous cell carcinoma by regulating miR‐200b/Kindlin‐2 axis. Cell Death Dis. 2017;8(6):e2888. 28640252 10.1038/cddis.2017.245 PMC5520904

[19] Zhang CY , Li RK , Qi Y , Li XN , Yang Y , Liu DL , et al. Upregulation of long noncoding RNA SPRY4‐IT1 promotes metastasis of esophageal squamous cell carcinoma via induction of epithelial‐mesenchymal transition. Cell Biol Toxicol. 2016;32(5):391–401. 27250657 10.1007/s10565-016-9341-1

[20] Dai F , Mei L , Meng S , Ma Z , Guo W , Zhou J , et al. The global expression profiling in esophageal squamous cell carcinoma. Genomics. 2017;109(3–4):241–50. 28442363 10.1016/j.ygeno.2017.04.005

[21] Chen S , Wu DD , Sang XB , Wang LL , Zong ZH , Sun KX , et al. The lncRNA HULC functions as an oncogene by targeting ATG7 and ITGB1 in epithelial ovarian carcinoma. Cell Death Dis. 2017;8(10):e3118. 29022892 10.1038/cddis.2017.486 PMC5682654

[22] Guo J , Ma J , Zhao G , Li G , Fu Y , Luo Y , et al. Long noncoding RNA LINC0086 functions as a tumor suppressor in nasopharyngeal carcinoma by targeting miR‐214. Oncol Res. 2017;25(7):1189–97. 28245169 10.3727/096504017X14865126670075 PMC7841018

[23] Guo Q , Qian Z , Yan D , Li L , Huang L . LncRNA‐MEG3 inhibits cell proliferation of endometrial carcinoma by repressing notch signaling. Biomed Pharmacother. 2016;82:589–94. 27470401 10.1016/j.biopha.2016.02.049

[24] Wang W , Zhu Y , Li S , Chen X , Jiang G , Shen Z , et al. Long noncoding RNA MALAT1 promotes malignant development of esophageal squamous cell carcinoma by targeting beta‐catenin via Ezh2. Oncotarget. 2016;7(18):25668–82. 27015363 10.18632/oncotarget.8257 PMC5041935

[25] Xie F , Liu H , Zhu YH , Qin YR , Dai Y , Zeng T , et al. Overexpression of GPR39 contributes to malignant development of human esophageal squamous cell carcinoma. BMC Cancer. 2011;11:86. 21352519 10.1186/1471-2407-11-86 PMC3053269

[26] Ye Y , Xu Y , Lai Y , He W , Li Y , Wang R , et al. Long non‐coding RNA cox‐2 prevents immune evasion and metastasis of hepatocellular carcinoma by altering M1/M2 macrophage polarization. J Cell Biochem. 2018;119(3):2951–63. 29131381 10.1002/jcb.26509

[27] Kudo‐Saito C , Shirako H , Takeuchi T , Kawakami Y . Cancer metastasis is accelerated through immunosuppression during snail‐induced EMT of cancer cells. Cancer Cell. 2009;15(3):195–206. 19249678 10.1016/j.ccr.2009.01.023

[28] Zhang C , Ma Q , Shi Y , Li X , Wang M , Wang J , et al. A novel 5‐fluorouracil‐resistant human esophageal squamous cell carcinoma cell line Eca‐109/5‐FU with significant drug resistance‐related characteristics. Oncol Rep. 2017;37(5):2942–54. 28393186 10.3892/or.2017.5539

[29] Kang M , Ren M , Li Y , Fu Y , Deng M , Li C . Exosome‐mediated transfer of lncRNA PART1 induces gefitinib resistance in esophageal squamous cell carcinoma via functioning as a competing endogenous RNA. J Exp Clin Cancer Res. 2018;37(1):171. 30049286 10.1186/s13046-018-0845-9 PMC6063009

[30] Jin Y , Wu D , Yang W , Weng M , Li Y , Wang X , et al. Hepatitis B virus x protein induces epithelial‐mesenchymal transition of hepatocellular carcinoma cells by regulating long non‐coding RNA. Virol J. 2017;14(1):238. 29258558 10.1186/s12985-017-0903-5 PMC5735895

[31] Shi X , Chen Z , Hu X , Luo M , Sun Z , Li J , et al. AJUBA promotes the migration and invasion of esophageal squamous cell carcinoma cells through upregulation of MMP10 and MMP13 expression. Oncotarget. 2016;7(24):36407–18. 27172796 10.18632/oncotarget.9239 PMC5095009

[32] Feng F , Qiu B , Zang R , Song P , Gao S . Pseudogene PHBP1 promotes esophageal squamous cell carcinoma proliferation by increasing its cognate gene PHB expression. Oncotarget. 2017;8(17):29091–100. 28404970 10.18632/oncotarget.16196 PMC5438715

[33] Lan X , Liu X . LncRNA SNHG1 functions as a ceRNA to antagonize the effect of miR‐145a‐5p on the down‐regulation of NUAK1 in nasopharyngeal carcinoma cell. J Cell Mol Med. 2019;23(4):2351–2361. 29575772 10.1111/jcmm.13497 PMC6434074

[34] Xin Z , Song X , Jiang B , Gongsun X , Song L , Qin Q , et al. Blocking FGFR4 exerts distinct anti‐tumorigenic effects in esophageal squamous cell carcinoma. Thorac Cancer. 2018;9:1687–98. 30267473 10.1111/1759-7714.12883 PMC6275831

[35] Hong Y , He H , Sui W , Zhang J , Zhang S , Yang D . Long non‐coding RNA H1 promotes cell proliferation and invasion by acting as a ceRNA of miR138 and releasing EZH2 in oral squamous cell carcinoma. Int J Oncol. 2018;52(3):901–12. 29344674 10.3892/ijo.2018.4247

[36] Zang W , Wang T , Wang Y , Chen X , du Y , Sun Q , et al. Knockdown of long non‐coding RNA TP73‐AS1 inhibits cell proliferation and induces apoptosis in esophageal squamous cell carcinoma. Oncotarget. 2016;7(15):19960–74. 26799587 10.18632/oncotarget.6963 PMC4991431

[37] Pan Y , Qin T , Yin S , Zhang X , Gao X , Mu L . Long non‐coding RNA UC001kfo promotes hepatocellular carcinoma proliferation and metastasis by targeting alpha‐SMA. Biomed Pharmacother. 2017;87:669–77. 28088733 10.1016/j.biopha.2017.01.018

[38] Feng YF , Lei YY , Lu JB , Xi SY , Zhang Y , Huang QT , et al. RIT1 suppresses esophageal squamous cell carcinoma growth and metastasis and predicts good prognosis. Cell Death Dis. 2018;9(11):1085. 30348939 10.1038/s41419-018-0979-x PMC6197279

[39] Qin Y , Zhang Y , Tang Q , Jin L , Chen Y . SQLE induces epithelial‐to‐mesenchymal transition by regulating of miR‐133b in esophageal squamous cell carcinoma. Acta Biochim Biophys Sin (Shanghai). 2017;49(2):138–48. 28069586 10.1093/abbs/gmw127

[40] Thiery JP , Acloque H , Huang RYJ , Nieto MA . Epithelial‐mesenchymal transitions in development and disease. Cell. 2009;139(5):871–90. 19945376 10.1016/j.cell.2009.11.007

[41] Upadhyay P , Gardi N , Desai S , Chandrani P , Joshi A , Dharavath B , et al. Genomic characterization of tobacco/nut chewing HPV‐negative early stage tongue tumors identify MMP10 asa candidate to predict metastases. Oral Oncol. 2017;73:56–64. 28939077 10.1016/j.oraloncology.2017.08.003 PMC5628952

[42] Zhang W , Hong R , Xue L , Ou Y , Liu X , Zhao Z , et al. Piccolo mediates EGFR signaling and acts as a prognostic biomarker in esophageal squamous cell carcinoma. Oncogene. 2017;36(27):3890–902. 28263981 10.1038/onc.2017.15

[43] Zhang H , Luo H , Hu Z , Peng J , Jiang Z , Song T , et al. Targeting WISP1 to sensitize esophageal squamous cell carcinoma to irradiation. Oncotarget. 2015;6(8):6218–34. 25749038 10.18632/oncotarget.3358 PMC4467433

