# Supplementary material for: Downregulation of long non‐coding RNA LOC101928477 correlates with tumor progression by regulating the epithelial‐mesenchymal transition in esophageal squamous cell carcinoma
Source: Thorac Cancer. 2021 Mar 13;12(9):1303–11. doi: 10.1111/1759-7714.13858 (PMC8088935; doi:10.1111/1759-7714.13858)
Supplement: Supplementary file 1 — Figure S1 Additional experiments using ESCC specimens and ESCC cells. (A) ESCC tissue showed higher MMP‐10 and Ki‐67 expression when compared with paired normal tissue. (B) Western blot analyses showed that the protein expression level of MMP‐10 in ESCC cells (especially EC109 and EC9706) was significantly higher than that of the normal human esophageal epithelial cell line. (C) Plate colony formation assay showed more colonies in LOC101928477‐knockdown group. (D‐E) LOC101928477‐knockdown increased the wound‐healing capacity of ESCC cells. Magnification: 100x. [file TCA-12-1303-s001.docx]

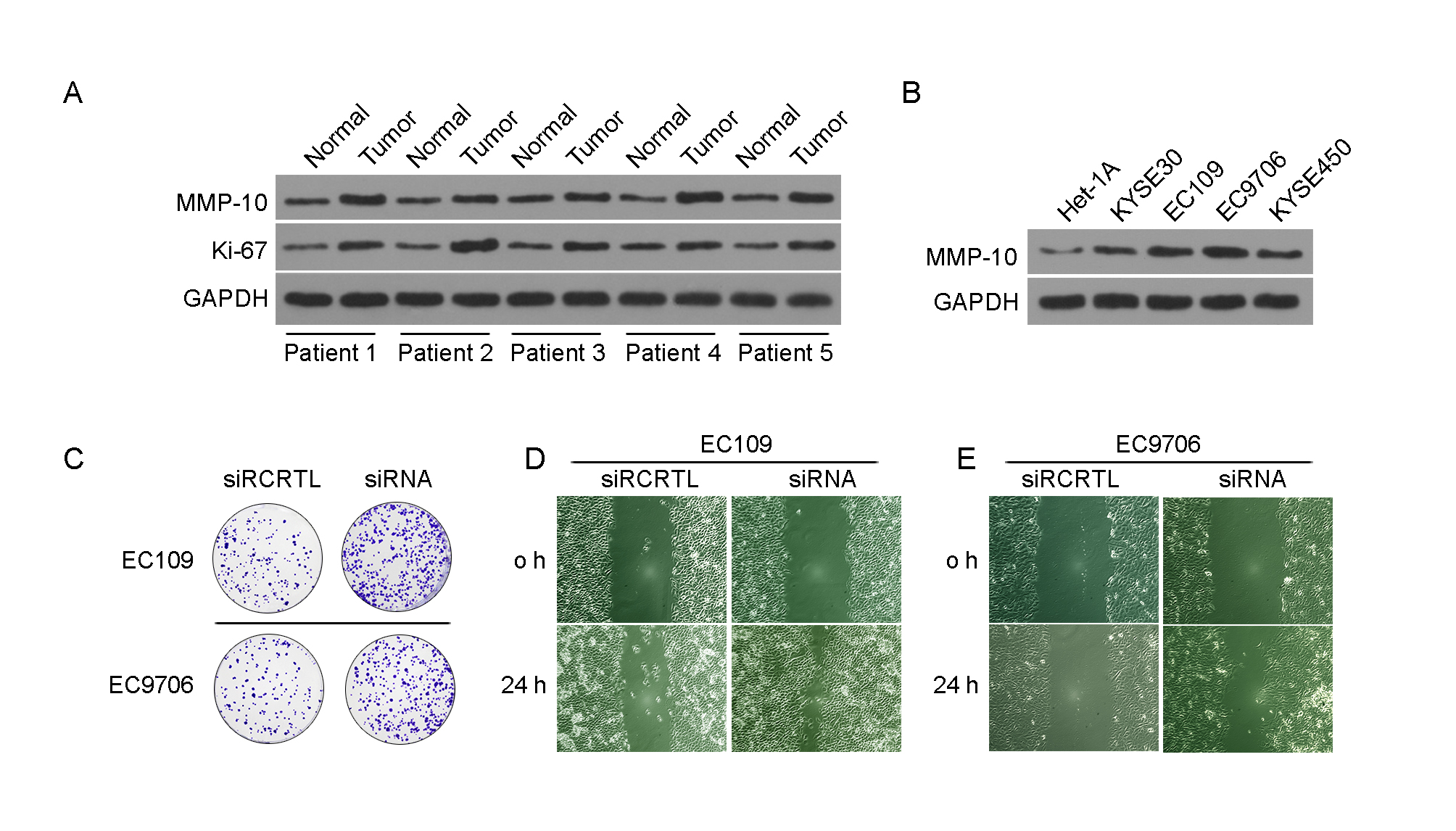


*Fig. S1. Additional experiments using ESCC specimens and ESCC cells.* (A) ESCC tissue showed higher MMP-10 and Ki-67 expression when compared with paired normal tissue. (B) Western blot analyses showed that the protein expression level of MMP-10 in ESCC cells (especially EC109 and EC9706) was significantly higher than that of the normal human esophageal epithelial cell line. (C) Plate colony formation assay showed more colonies in LOC101928477-knockdown group. (D-E) LOC101928477-knockdown increased the wound-healing capacity of ESCC cells. Magnification: 100x.
